# IV/IT hUC-MSCs Infusion in RRMS and NMO: A 10-Year Follow-Up Study

**DOI:** 10.3389/fneur.2020.00967

**Published:** 2020-09-08

**Authors:** Zhengjuan Lu, Lin Zhu, Zhuo Liu, Jiayong Wu, Yun Xu, Cun-Jin Zhang

**Affiliations:** ^1^Department of Neurology of Nanjing Drum Tower Hospital, Medical School and the State Key Laboratory of Pharmaceutical Biotechnology, Nanjing University, Nanjing, China; ^2^Department of Neurology, Institute of Brain Sciences, Nanjing University, Nanjing, China; ^3^Jiangsu Key Laboratory for Molecular Medicine, Medical School of Nanjing University, Nanjing, China; ^4^Jiangsu Province Stroke Center for Diagnosis and Therapy, Nanjing, China; ^5^Nanjing Neuropsychiatry Clinic Medical Center, Nanjing, China

**Keywords:** human umbilical cord–derived mesenchymal stem cells, neuromyelitis optica, relapse remitting multiple sclerosis, safety, long-term follow-up

## Abstract

**Background:** Stem cell transplantation is emerging as a potential therapeutic strategy in several autoimmune diseases. However, the safety and feasibility of long-term combined intravenous (IV) and intrathecal (IT) administration of hUC-MSCs in relapse remitting multiple sclerosis (RRMS) and neuromyelitis optica (NMO) is largely unknown.

**Objectives:** In this study, we followed up the long-term safety and feasibility of combined IV and IT human umbilical cord mesenchymal stem cells (hUC-MSCs) transplantation in patients with RRMS and NMO.

**Methods:** Five NMO patients and 5 RRMS patients were treated intravenously (4 times) and intrathecally (3 times) over a 21-day period with low-dose allogeneic umbilical cord blood–derived MSCs. All of the patients were monitored regularly by an investigator in a blinded manner to access the Expanded Disability Status Scale, MRI characteristics, and adverse events every 3 months within 12 months and once every year thereafter for 10 years after transplantation.

**Results:** During the long-term follow-up, our data suggested that combined IV and IT administration of hUC-MSCs transplantation is safe and feasible. None of the intolerant adverse events, such as tumor formation and peripheral organ/tissue disorders, were observed throughout the 10-year follow-up.

**Conclusions:** These data suggest that combined intravenous and intrathecal low-dose hUC-MSCs transplantation is safe and feasible in RRMS and NMO patients in the long term. The conclusion requires confirmation by future clinical trials in a larger cohort.

## Introduction

Multiple sclerosis (MS) is a chronic, immune-mediated, demyelinating disorder of the central nervous system (CNS) (1). The eventual demyelination and secondary axonal degeneration can cause damage in sensation, mobility, balance, sphincter function, vision, and cognition. The disease predominantly affects individuals in their early adult lives. There are about 2.3 million people living with multiple sclerosis globally, which crucially increases the burden of family and society. The first-line treatments mainly depend on steroids for acute episodes and disease-modifying drugs (DMDs) for chronic stage; however, these therapeutic approaches share some common characteristics, such as suppressed immune system and compromised host defense to bacterial and virus infection. In addition, some deadly events like progressive multifocal leukoencephalopathy were reported after some long-term DMD treatment. It is still required and necessary to investigate and develop safer and better tolerated therapeutic strategy for MS.

Neuromyelitis optica (NMO), characterized by recurrent episodes of longitudinally extensive transverse myelitis and/or optica neuritis, is another autoimmune inflammatory demyelinating disorder of CNS, which is distinct from MS in both mechanism of action and clinic treatment after discovery of the serum aquaporin 4 antibody (AQP4-Ab). AQP4 is the main water channel of the CNS that is dominantly expressed on astrocytes (2). AQP4-Ab was found in around 80% of NMO patients, whereas GFAP-Ab and MOG-Ab were also identified as the pathogenic factor in some NMO patients. Recent studies have extended the phenotype of NMO, with MRI revealing the clinically relevant abnormalities within the brain involving the diencephalon, area postrema, corpus callosum, hemispheric white matter, and corticospinal tracts. Treatments for temporary flare-ups include steroids, intravenous immunoglobulin therapy (IVIG), B-cell depletion, and plasma exchange. The management of NMO recurrence was mainly dependent on the immunosuppressive approaches, which caused a variety of adverse reactions including opportunistic infection, leukopenia, and organ damage.

Stem cell–based therapies are considered promising strategies for these inflammatory demyelinating diseases (3–6). Experimental evidence in preclinical models of MS suggests that mesenchymal stem cells (MSCs) from different sources such as bone marrow (BM), umbilical, adipose tissue, and autologous hemopoietic system are capable of modifying both immune reactions and endogenous repair mechanisms (7). Autologous hemopoietic stem-cell transplantation has achieved impressive efficiency and significantly enhanced improvement than DMDs in MS patients in a phase III large-scale clinical trial (3); however, allogeneic umbilical cord stem cell transplantation in NMO and MS patients still lacks a large cohort study, and the safety issue is only partially known in short-term but not long-term studies. In addition, almost all of the stem cells were transferred intravenously with very limited cells passing the blood–brain barrier. The feasibility of combined intravenous and intrathecal administration in NMO and MS is not completely understood. Although human umbilical cord–derived stem cells (hUC-MSCs) have unique advantages owing to their convenient sampling, easy amplification, and non-antigenicity, its feasibility and safety are still uncertain (8–11). To date, previous clinical trials with stem cell transplantation referred to short-term follow-ups were mostly monitored <5 years. Here, we present the results of a 10-year follow-up of 10 patients who suffered from relapse remitting multiple sclerosis (RRMS) (5 patients) or NMO (5 patients) after hUC-MSCs transplantation and did not observe any severe adverse events, suggesting hUC-MSCs transplantation may be safe and feasible in MS and NMO patients.

## Materials and Methods

### Patients

The present study was the follow-up of a previous clinical trial, which was approved by the Ethics Committee of the Affiliated Drum Tower Hospital of Nanjing University Medical school (ClinicalTrials.gov Identifier: NCT b136424). Ten patients, with ages ranging from 19 to 59 years, were recruited from the Department of Neurology of the affiliated Drum Tower Hospital of Nanjing University Medical School, between April 2009 and December 2010. All patients provided written informed consent. The enrollment criteria were (1) diagnosed as clinical RRMS according to McDonald criteria; diagnosed as NMO, beyond the optic nerves and spinal cord damage, NMO-IgG seropositive status is essential; (2) aged 18–65 years; (3) duration of disease longer than 4 years and Expand Disability Status Scale (EDSS) score 2.5–8.5; and (4) failure to respond to the currently available and registered agents for MS and NMO. The exclusion criteria were (1) participation in other clinical trials during 3 months before the trial; (2) severe cardiac, hepatic, and renal failure or any other disease that may influence the result of the study; (3) pregnant or possibly pregnant; (4) significant cognitive decline or inability to understand the treatment protocol or sign the informed consent; and (5) allergies. The baseline characteristics of the patients are described in Table 1.

**Table 1:** Characteristics of NMO and MS patients.

**Variable**	**NMO patients**					**MS patients**				
	**Case 1**	**Case 2**	**Case 3**	**Case 4**	**Case 5**	**Case 6**	**Case 7**	**Case 8**	**Case 9**	**Case 10**
Age at onset, years	30	19	29	14	35	52	52	26	25	22
Age range at enrollment, years	30–35	40–45	35–40	15–20	35–40	55–60	55–60	40–45	30–35	25–30
Relapses since disease onset, *n*	5	8	5	3	3	21	3	5	12	3
Abnormal visual evoked potential	Yes	Yes	Yes	Yes	Yes	Yes	Yes	Yes	Yes	Yes
Previous therapy	CS IVIG CTX	CS IVIG	CS	CS IFNβ	CS	CS IVIG	CS IVIG	CS IFNβ AZA	CS IFNβ	CS

*CS, corticosteroid; IVIG, intravenous immunoglobulin; CTX, cyclophosphamide; AZA, azathioprine*.

### hUC-MSCs Preparation and Transfusions

hUC-MSCs were provided by the Clinical Stem Cell Center of Jiangsu Province (Jiangsu Beike Bio-Technology). The exact procedures for hUC-MSCs preparation were described as follows. The UCs were carefully washed in 1 × PBS containing antibiotics and were cut into small pieces (1–2 mm) and moved into DMEM medium, containing human basic fibroblast growth factor (bFGF), epidermal growth factor (EGF), platelet-derived growth factor-BB (PDGF-BB), and transforming growth factor (TGF)-1. After incubation in a humidified incubator containing 5% CO_2_ and 95% oxygen at 37°C for about 10 days, well-developed colonies of fibroblast-like cells could be found and subculture could proceed. All hUC-MSCs used for transfer were passage 2 with viability > 95%. All the hUC-MSCs were CD73, CD90, CD105 positive and CD45, CD34, CD79, CD14 negative. Every patient received four infusions: on day 0, 40 ml of hUC-MSCs (4 × 10^7^ cells) were intravenously infused and then 20 ml (2 × 10^7^ cells) were injected intravenously combined with 1 ml (2 × 10^7^ cells) intrathecally on days 7, 14, and 21, respectively. In other words, all participants completed four times infusions (4 × 10^7^, 2 × 10^7^, 2 × 10^7^, 2 × 10^7^ hUC-MSCs were administrated intravenously to patients on day 0, 7, 14, and 21, respectively, and 2 × 10^7^, 2 × 10^7^, 2 × 10^7^ hUC-MSCs were administrated intrathecally to patients on day 7, 14, and 21, respectively) during the remitting disease stage. None of the immune-modified medicines were applied during hUC-MSCs treatment.

### Clinical Evaluation of Efficacy and Safety

The clinical evaluation was assessed by an investigator who is blinded to this study. EDSS score and occurrence of relapses were obtained at baseline and follow-up for 10 years. The relapse was defined by identification of new lesion on brain/spinal cord as determined by MRI, as well as having new symptoms or an increase in existing symptoms. MRI were performed at the time of recruitment, 6 months after transplantation and each relapse. The examination were performed by two independent radiologists blind to the intervention. Follow-up visits were scheduled every 3 months within 1 year and once per year thereafter. Short-term and long-term adverse events were recorded during the follow-up period. Possible short-term adverse events included allergic reactions (fever, tachycardia, rash), respiratory failure, local complications (headache, dizziness, fatigue, infection related to lumbar puncture) whereas monitored long-term adverse events included tumor formation and aberrant connection (newly diagnosed myoclonus, ataxia) that were possibly related to MSC treatment. The evaluation was performed by a neurologist in a blinded manner.

## Results

### Baseline Characteristics, Treatments of Patients, and Follow-Up

**Figure 1:**
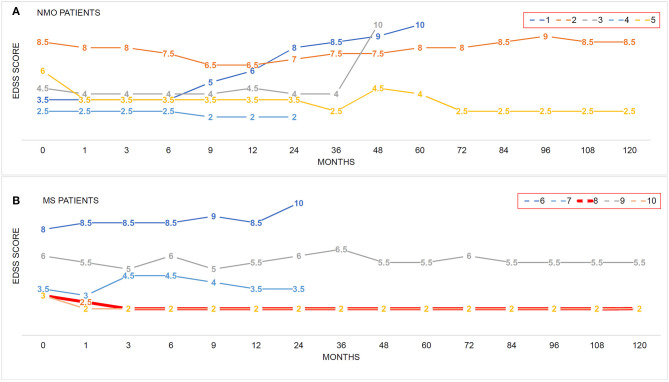
The impacts of hUC-MSC transplantation on disease severity in NMO and MS. **(A,B)** EDSS score was used to evaluate the disease severity before and after hUC-MSC transplantation. Each colored line indicates one case. Number in each line indicates the EDSS score at these time points. EDSS = 10 means death of the patients.

**Figure 2:**
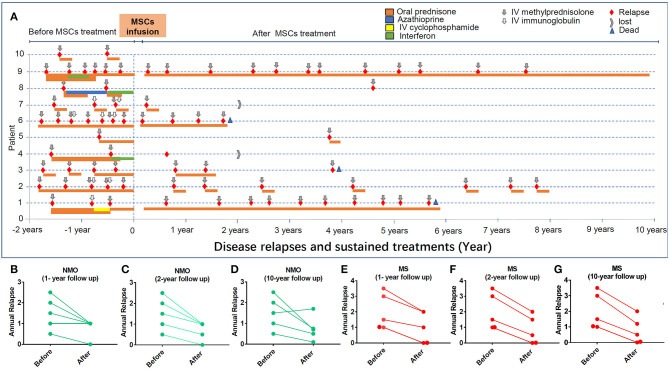
The impacts of hUC-MSC transplantation on disease relapse in NMO and MS. **(A)** Each relapse was colored with red. Gray arrow indicates the remitting treatments. All the medicines applied before and after hUC-MSCs are indicated. **(B–D)** The relapse of NMO disease in 1, 2, and 10 years after hUC-MSC transplantation. **(E–G)** The relapse of MS disease in 1, 2, and 10 years after hUC-MSC transplantation.

Because both NMO and MS diseases were affected more in women, all of the patients enrolled in the current follow-up study were female and the mean disease duration of participants was 6.4 years. Patients 1–5 had a diagnosis of NMO, whose mean EDSS score was 5, and patients 6–10 were diagnosed RRMS with mean EDSS score of 4.7 at baseline (Table 1). Patients were followed up at 1 month and every 3 months within the first year, every 1 year thereafter. Two participants were lost to follow-up after the 2-year visit and three patients died as a result of disease progression. The others were followed up to 10 years (Figures 1, 2).

### hUC-MSC Transplantation by Combined IV and IT Administration Is Feasible in Both NMO and MS Patients

EDSS scores and disease relapse were evaluated by an investigator who was blinded to the study to follow up the changes of disease severity (Figures 1A,B). One month after the hUC-MSCs transplantation, EDSS score of all five NMO cases (patients 1–5, Figure 1A) and four MS cases (patients 7–10, Figure 1B) were reduced, and one MS case (patient 6, Figure 1B) was increased. However, these effects could also be a possible result of resolution of disease itself. At 3 and 9 months after the transplantation, one more MS case (patient 7, Figure 1B) and one NMO case (patient 1, Figure 1A) have shown increased disease severity. One of the key shared characteristics of NMO and RRMS is the frequent disease relapse which was also investigated in the current study in a blinded manner to evaluate the safety of hUC-MSCs. Compared with the annual relapse occurrence rate 2 years before the hUC-MSC transplantation, all of the NMO and MS cases demonstrated reduced annual relapse occurrence in 1 and 2 years after the transplantation (Figures 2A–C,E,F). During the long-term follow-up, 9 of 10 patients (except NMO patient 1) showed potentially diminished relapse occurrence without any side effect–related complaints (Figures 2A,D,G), which suggest the feasibility of hUC-MSCs in both NMO and MS.

### hUC-MSC Transplantation Was Well-Tolerated in the Long Term

**Figure 3:**
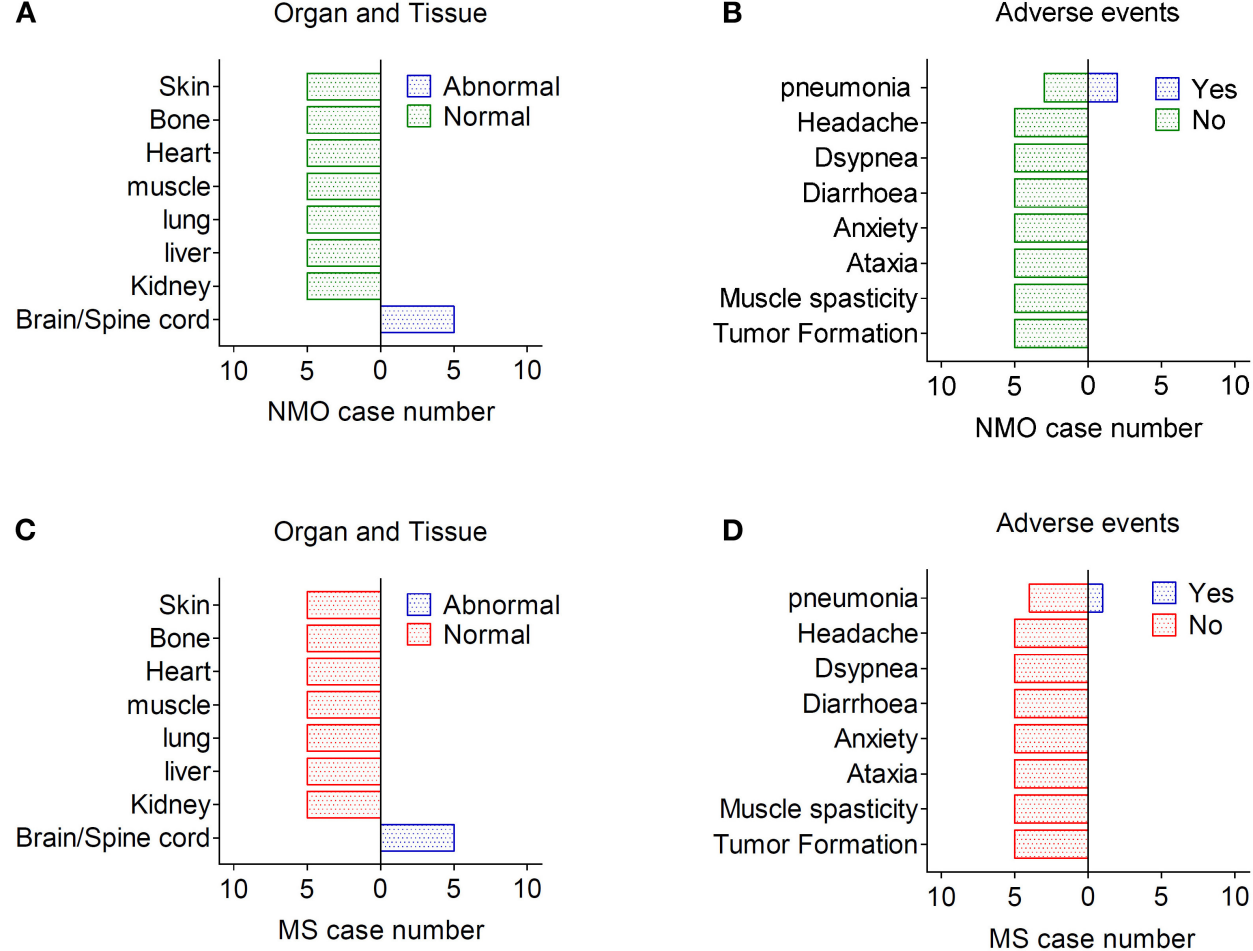
Adverse effects after hUC-MSC transplantation in NMO and MS. **(A,B)** Organ, tissue, and other presentative adverse events in NMO patients after hUC-MSC transplantation. **(C,D)** Organ, tissue, and other presentative adverse events in MS patients after hUC-MSC transplantation.

The long-term effects of hUC-MSCs were analyzed by investigation of organ/tissue function and possible adverse events. Although tumor formation and other side effects were reported previously in few cases after stem cell transplantation, (12, 13) we did not observe any case that developed tumors as well as other organ disorders throughout the follow-up in our study, although partial demyelination lesions were still found in the brain and spinal cord of NMO/MS patients (Figures 3A–D). Three patients died as demyelinating disease progress rapidly with the common complications of pneumonia and pulmonary embolism after 24 months; however, these infectious events may not be related to the hUC-MSCs transplantation. Some other possible short-term and long-term adverse events, including allergic reactions, local and systemic complications, and aberrant connections (newly diagnosed ataxia, myoclonus), were neither observed during follow-up nor complained by patients in our study, suggesting hUC-MSCs implantation is safe and feasible in NMO and MS (Figures 3A–D).

## Discussion

MSCs have emerged as a valuable resource for cell-based therapies because of their immunomodulatory and neural regenerative effects. There have been more than 20 clinical trials of different kinds of MSC transplantation for demyelinating autoimmune diseases including MS and NMO in recent 15 years, some of which had got promising results (14–16). However, all the previous work regarding of hUC-MSCs in NMO and MS were followed up <5 years, and there is a lack of safety evidence of hUC-MSCs therapy in the long term in NMO and MS (14–16). MSC-based therapies are considered to be relatively safe at short term based on the results of those who completed clinical trials (14–16). However, the long-term safety of MSCs is still lacking evidences. We provided the important data of long-term follow-up of hUC-MSCs transplantation via combined IV and IT administration in MS and NMO patients and did not observe neoplasm formation and any other key organ injury/dysfunction according during the 10-year follow-up.

Transplantation of stem cell by intravenous approach is common in clinic; however, most of the cells were trapped in the peripheral with few cell migration to the CNS. Although intravenous transfer of stem cell obtained certain benefit clinically, one of the critical questions is whether the stem cell in the CNS could further enhance the tissue repair and brain damage. One of the hypotheses is that combined intravenous and intrathecal hUC-MSCs transplantation may improve the therapeutic effects of stem cells, which requires assessing the safety and feasibility of combined approach. Allogeneic hUC-MSCs transplantation has the possibility to trigger short-time abnormal immune responses, such as allergy. To reduce the potential risks during the cell transplantation as much as possible, we separate the intravenous infusion from intrathecal infusion. Strategically, three times of intravenous infusion were applied first followed with three times of intrathecal infusion in few weeks.

NMO and MS often have significant degenerative characteristics and a poor prognosis (1, 2). In the current study, 3 patients out of 10 died during the course of this study, which may be a result of disease progression. The common feature of three patients who died is rapid disease progression at a specific time point with a finial EDSS score at 10, a severe clinical score directly resulting in the death. At the same time, the EDSS score of the two cases developed to 10 in 1 year and the other one developed to EDSS score 10 in 3.5 years. Of note, none of the stem cell transplantation–associated side effects (such as tumors, peripheral organ disorders) were observed in these three patients, suggesting the death was an outcome of disease progression but not the consequence of stem cell treatment. Although all three patients received combined treatments, including oral prednisone and IV methylprednisolone, the diseases progression was not mitigated.

The major purpose of this study is to evaluate the safety of the combined IT/IV hMSCs treatment in the long term. Because none of the placebo groups were included in the current study, it is insufficient to make any conclusions on treatment efficiency although the relapse frequency has a reduced trend after combined IT/IV hMSCs treatments. In addition, the treatment, such as IVIG, is not an effective therapy in MS as evidenced previously, which may not impact the conclusion of the safety generated in this study. Although the complicated treatment after the relapse may compromise the immune system and increase the risk of organ disorders and tumor generation, none of the severe side effects were observed during the long-term follow-up, suggesting the combined IT/IV hMSCs treatment is potentially safe.

Transformation to cancer is a common concern and critical possible safety issue after stem cell treatment (17–19) Some studies have shown *ex vivo* expanded hMSCs were not prone to undergo neoplastic transformation even after exposure to supramaximal doses of ionizing radiation and starvation culture (20). Furthermore, Subramanian et al. (18) reported hUC-MSCs even possessed the unique property of being anti-tumorigenic because they did not transform to tumor-associated fibroblasts in the presence of breast and ovarian cancer cells unlike bone marrow-derived MSCs, which suggested hUC-MSCs are possibly safe for clinical application; however, the long-term effects were still unknown. Our study provided more supporting evidences for safety of hUC-MSCs in clinic in a long time period.

Although some short-term adverse events were reported, we did not observe any transplantation-associated side event in both the short term and long term, which may be due to the difference in total cell number and approaches of transplantation. The adverse events were thought to be in a dose-dependent fashion, that a dose of 1 × 10^8^ cells was related to more low back and leg pain (17). Based on a dose of 1 × 10^6^ MSCs/kg body weight in systemic lupus erythematosus following hUC-MSC transplantation, (21) we used a lower total dose of 4 × 10^7^ MSCs. We elected to administer cells intravenously combined intrathecally to ensure enough cells enter the CNS because the lungs may trap many cells following IV injection. The results from the current study suggest that a lower cell number of transplantations was possibly associated with reduced side effects, which requires further confirmation in a larger cohort study.

Although the most common approach of stem cells transplantation in MS/NMO patients was intravenous, (3, 5) combined intravenous and intrathecal administration in clinic is rare due to the complexities. Allogeneic umbilical cord stem cells have unique advantages, including ease of collection, lower immunogenicity, and faster self-renewal compared with other sources, such as bone marrow–derived MSCs, which allow hUC-MSCs to be an attractive and potential therapy in NMO/MS.

## Conclusion

In summary, this long-term follow-up study suggests that hUC-MSC transplantation may be safe and well-tolerated in MS and NMO. However, because of the limitation in the current study, such as lack of comparison group and small sample size, future clinical trials with a larger cohort and comparison group are required to confirm the conclusion generated in this study.

## Data Availability Statement

The datasets generated for this study are available on request to the corresponding author.

## Ethics Statement

The studies involving human participants were reviewed and approved by Ethics Committee of the Affiliated Drum Tower Hospital of Nanjing University Medical school. The patients/participants provided their written informed consent to participate in this study.

## Author Contributions

C-JZ designed and conceived this study. YX provided key suggestions. ZLu finished the majority of the experiments and analysis. LZ, ZLi, and JW helped with the experiments. All authors contributed to the article and approved the submitted version.

## Conflict of Interest

The authors declare that the research was conducted in the absence of any commercial or financial relationships that could be construed as a potential conflict of interest.

## References

[1] Thompson AJ Baranzini SE Geurts J Hemmer B Ciccarelli O. Multiple sclerosis. Lancet. (2018) 391:1622–36. 10.1016/S0140-6736(18)30481-1 29576504

[2] Bruscolini A Sacchetti M La Cava M Gharbiya M Ralli M Lambiase A. Diagnosis and management of neuromyelitis optica spectrum disorders - An update. Autoimmun Rev. (2018) 17:195–200. 10.1016/j.autrev.2018.01.001 29339316

[3] Burt RK Balabanov R Burman J Sharrack B Snowden JA Oliveira MC. Effect of nonmyeloablative hematopoietic stem cell transplantation vs continued disease-modifying therapy on disease progression in patients with relapsing-remitting multiple sclerosis: a randomized clinical trial. JAMA. (2019) 321:165–74. 10.1001/jama.2018.18743 30644983 PMC6439765

[4] Muraro PA Martin R Mancardi GL Nicholas R Sormani MP Saccardi R Autologous haematopoietic stem cell transplantation for treatment of multiple sclerosis. Nat Rev Neurol. (2017) 13:391–405. 10.1038/nrneurol.2017.81 28621766

[5] Atkins HL Bowman M Allan D Anstee G Arnold DL Bar-Or A. Immunoablation and autologous haemopoietic stem-cell transplantation for aggressive multiple sclerosis: a multicentre single-group phase 2 trial. Lancet. (2016) 388:576–85. 10.1016/S0140-6736(16)30169-6 27291994

[6] Scolding NJ Pasquini M Reingold SC Cohen JA. Cell-based therapeutic strategies for multiple sclerosis. Brain. (2017) 140:2776–96. 10.1093/brain/awx154 29053779 PMC5841198

[7] Liu R Zhang Z Lu ZJ Borlongan C Pan J Chen JH. Human umbilical cord stem cells ameliorate experimental autoimmune encephalomyelitis by regulating immunoinflammation and remyelination. Stem Cells Dev. (2013) 22:1053–62. 10.1089/scd.2012.0463 23140594

[8] Donders R Bogie JFJ Ravanidis S Gervois P Vanheusden M Marée R. Human wharton's jelly-derived stem cells display a distinct immunomodulatory and proregenerative transcriptional signature compared to bone marrow-derived stem cells. Stem Cells Dev. (2018) 27:65–84. 10.1089/scd.2017.0029 29267140

[9] Yang HNY Sun JHS Wang F Li Y Bi JZ Qu TY. Umbilical cord-derived mesenchymal stem cells reversed the suppressive deficiency of T regulatory cells from peripheral blood of patients with multiple sclerosis in a co-culture - a preliminary study. Oncotarget. (2016) 7:72537–45. 10.18632/oncotarget.12345 27705922 PMC5341927

[10] Meng M Liu Y Wang W Wei C Liu F Du Z. Umbilical cord mesenchymal stem cell transplantation in the treatment of multiple sclerosis. Am J Transl Res. (2018) 10:212–23. 29423006 PMC5801359

[11] Riordan NH Morales I Fernández G Allen N Fearnot NE Leckrone ME. Clinical feasibility of umbilical cord tissue-derived mesenchymal stem cells in the treatment of multiple sclerosis. J Transl Med. (2018) 16:57. 10.1186/s12967-018-1433-7 29523171 PMC5845260

[12] Zhao TB Zhang ZN Westenskow PD Todorova D Hu Z Lin TX. Humanized mice reveal differential immunogenicity of cells derived from autologous induced pluripotent stem cells. Cell Stem Cell. (2015) 17:353–9. 10.1016/j.stem.2015.07.021 26299572 PMC9721102

[13] Wood KJ Issa F Hester J. Understanding stem cell immunogenicity in therapeutic applications. Trends Immunol. (2016) 37:5–16. 10.1016/j.it.2015.11.005 26687737

[14] Connick P Kolappan M Crawley C Webber DJ Patani R Michell AW. Autologous mesenchymal stem cells for the treatment of secondary progressive multiple sclerosis: an open-label phase 2a proof-of-concept study. Lancet Neurol. (2012) 11:150–6. 10.1016/S1474-4422(11)70305-2 22236384 PMC3279697

[15] Lublin FD Bowen JD Huddlestone J Kremenchutzky M Carpenter A Corboy JR. Human placenta-derived cells (PDA-001) for the treatment of adults with multiple sclerosis: a randomized, placebo-controlled, multiple-dose study. Mult Scler Relat Disord. (2014) 3:696–704. 10.1016/j.msard.2014.08.002 25891548

[16] Cohen JA Imrey PB Planchon SM Bermel RA Fisher E Fox RJ. Pilot trial of intravenous autologous culture-expanded mesenchymal stem cell transplantation in multiple sclerosis. Mult Scler. (2018) 24:501–11. 10.1177/1352458517703802 28381130 PMC5623598

[17] Staff NP Madigan NN Morris J Jentoft M Sorenson EJ Butler G. Safety of intrathecal autologous adipose-derived mesenchymal stromal cells in patients with ALS. Neurology. (2016) 87:2230–4. 10.1212/WNL.0000000000003359 27784774 PMC5123559

[18] Subramanian A Shu-Uin G Kae-Siang N Gauthaman K Biswas A Choolani M. Human umbilical cord Wharton's jelly mesenchymal stem cells do not transform to tumor-associated fibroblasts in the presence of breast and ovarian cancer cells unlike bone marrow mesenchymal stem cells. J Cell Biochem. (2012) 113:1886–95. 10.1002/jcb.24057 22234854

[19] Wang Y Zhang Z Chi Y Zhang Q Xu F Yang Z. Long-term cultured mesenchymal stem cells frequently develop genomic mutations but do not undergo malignant transformation. Cell Death Dis. (2013) 4:e950. 10.1038/cddis.2013.480 24309937 PMC3877551

[20] Conforti A Starc N Biagini S Tomao L Pitisci A Algeri M. Resistance to neoplastic transformation of *ex-vivo* expanded human mesenchymal stromal cells after exposure to supramaximal physical and chemical stress. Oncotarget. (2016) 7:77416–29. 10.18632/oncotarget.12678 27764806 PMC5363595

[21] Sun LY Wang DD Liang J Zhang HY Feng XB Wang H. Umbilical cord mesenchymal stem cell transplantation in severe and refractory systemic lupus erythematosus. Arthritis Rheum. (2010) 62:2467–75. 10.1002/art.27548 20506343

